# Temporal single-cell sequencing analysis reveals that GPNMB-expressing macrophages potentiate muscle regeneration

**DOI:** 10.1038/s12276-025-01467-4

**Published:** 2025-06-09

**Authors:** Yu-Fan Chen, Chien-Wei Lee, Yi-Shuan J. Li, Wei-Ting Lin, Hsiao-Yun Chen, Yu-Chuan Chen, Chia-Hao Lin, Jennifer Hui-Chun Ho, Li-Fan Lu, Shu Chien, Oscar Kuang-Sheng Lee

**Affiliations:** 1 Biomedical Technology and Device Research Laboratories, Industrial Technology Research Institute, Hsinchu, Taiwan; 2 Yang Ming Regenerative Therapeutics Co., Ltd., Taichung, Taiwan; 3 Translational Cell Therapy Center, China Medical University Hospital, Taichung, Taiwan; 4 Institute of Engineering in Medicine, University of California San Diego, La Jolla, CA USA; 5 Department of Bioengineering, University of California San Diego, La Jolla, CA USA; 6 Doctoral Degree Program of Translational Medicine, National Yang Ming Chiao Tung University and Academia Sinica, Taipei, Taiwan; 7 School of Biological Sciences, University of California San Diego, La Jolla, CA USA; 8 Department of Medical Research, Eye Center, China Medical University Hospital, China Medical University, Taichung, Taiwan; 9 Moores Cancer Center, University of California San Diego, La Jolla, CA USA; 10 Center for Microbiome Innovation, University of California San Diego, La Jolla, CA USA; 11 Department of Biotechnology Medicine, MacKay Memorial Hospital, Taipei, Taiwan

**Keywords:** Immunology, Cell biology

## Abstract

Macrophages play a crucial role in coordinating the skeletal muscle repair response, but their phenotypic diversity and the transition of specialized subsets to resolution-phase macrophages remain poorly understood. Here, to address this issue, we induced injury and performed single-cell RNA sequencing on individual cells in skeletal muscle at different time points. Our analysis revealed a distinct macrophage subset that expressed high levels of *Gpnmb* and that coexpressed critical factors involved in macrophage-mediated muscle regeneration, including *Igf1, Mertk* and *Nr1h3*. *Gpnmb* gene knockout inhibited macrophage-mediated efferocytosis and impaired skeletal muscle regeneration. Functional studies demonstrated that GPNMB acts directly on muscle cells in vitro and improves muscle regeneration in vivo. These findings provide a comprehensive transcriptomic atlas of macrophages during muscle injury, highlighting the key role of the GPNMB macrophage subset in regenerative processes. Our findings suggest that modulating GPNMB signaling in macrophages may represent a promising avenue for future research into therapeutic strategies for enhancing skeletal muscle regeneration.

## GPNMB macrophages drive muscle regeneration after injury

Skeletal muscle, a major tissue in our bodies, needs to heal well after injury. Researchers have found that a protein called GPNMB plays a key role in this process. Researchers used a mouse model to explore how GPNMB affects muscle repair. They injured the muscles of mice and observed changes in macrophages over time. They found that GPNMB levels increased in certain macrophages, which helped the muscle heal. They used techniques such as single-cell RNA sequencing to identify five types of macrophage involved in healing. One type, with high GPNMB levels, was crucial for muscle repair. When they removed GPNMB from mice, muscle healing was impaired. Adding GPNMB back improved healing by promoting the growth of new muscle cells. This study suggests that targeting GPNMB could facilitate the development of treatments for muscle injuries. This summary was initially drafted using artificial intelligence, then revised and fact-checked by the author.

## Introduction

Skeletal muscle is the most abundant human body tissue, accounting for 30–45% of body weight, and the maintenance of its integrity and homeostasis is of critical importance. The complete regeneration of skeletal muscle after injury requires coordinated communication among several distinct cell types, including immune cells, muscle stem cells (MuSCs), fibro-/adipogenic progenitors (FAPs), glial cells and vascular cells^1,2^. Tight control of signal integration in the injury-induced immune response has been shown to promote regeneration in several tissues, including the liver^3^, heart^4^ and skeletal muscle^5^. In the past, research on the role of macrophages in tissue regeneration has focused on their ability to phagocytose cellular debris. However, infiltrating macrophages can undergo a polarization shift toward an anti-inflammatory phenotype and exhibit various pro-regenerative functions. These functions include the remodeling of the extracellular matrix^6^ and the stimulation of MuSC proliferation^7^. Disturbances in macrophage function lead to impaired muscle regeneration^8^, highlighting the importance of these cells in the regeneration process. It has been suggested that, in mice, Ly6C^lo^ macrophages contribute to skeletal muscle^9,10^ and myocardial tissue regeneration^11^. The core genes expressed in Ly6C^lo^ macrophages include secretory cytokines and growth factors such as insulin-like growth factor 1 (IGF1)^12^, the growth differentiation factors GDF3^13^ and GDF15^14^, and transforming growth factor beta (TGF-β)^13,15^, which act in an anti-inflammatory manner and contribute to skeletal muscle regeneration.

Glycoprotein nonmetastatic melanoma protein B (GPNMB), which was initially identified as a regulator of tumor growth in melanoma with low metastatic potential^16^, is involved in the transendothelial migration of dendritic cells^17^. GPNMB also inhibits osteoclast differentiation by interacting with CD44 and inhibiting ERK activation^18^. In the brain, GPNMB is predominantly expressed in microglia, which serve as resident cells responsible for mediating inflammatory stimuli and neurodegeneration^19^. Furthermore, GPNMB expression is elevated in human liver samples from patients with hepatitis, cirrhosis and paracetamol intoxication, all of which are associated with inflammatory diseases, compared to samples from healthy controls. Notably, the deletion of GPNMB in mice led to a significant increase in the levels of inflammatory cytokines in macrophages, suggesting that GPNMB may suppress the transition of macrophages toward a proinflammatory state^20^. However, the precise biological roles of GPNMB in macrophages during skeletal muscle regeneration remain to be elucidated.

In this study, we used a cardiotoxin (CTX)-induced skeletal muscle injury model, which induces myofiber necrosis and provides a highly reproducible framework. This model allowed us to visualize temporal changes in macrophage subsets and analyze their roles in muscle regeneration. We discovered that the expression of GPNMB was upregulated in a sustained manner, reaching its highest level on day 3, in a specific macrophage subset that coexpresses critical factors involved in macrophage-mediated muscle regeneration. Furthermore, myeloid-specific *Gpnmb* knockdown impaired the regeneration of skeletal muscle. The extrinsic administration of recombinant GPNMB (rGPNMB) to injured mice promoted myogenesis by activating myocyte differentiation. Overall, our study describes unrecognized macrophage subsets involved in muscle regeneration and demonstrated that GPNMB acts on myocytes in vitro and promotes muscle regeneration in vivo.

## Materials and methods

### Animals

All animal experiments were approved by the Institutional Animal Care and Use Committee of China Medical University Hospital (license no. CMUIACUC-2023–061). Eight- to 12-week-old female C57BL/6J mice were provided by the National Laboratory Animal Center (Taiwan) and housed at the China Medical University Hospital Animal Center. Gpnmb^−/−^ mice were generated in cooperation with the Transgenic Core Facility at the Academia Sinica, Taiwan, using the CRISPR–Cas9 genome editing system. Two guide ribonucleoproteins (RNAs) targeting sequences flanking exons 2–6 of the *Gpnmb* gene were designed and combined with Cas9-RNPs for microinjection into fertilized C57BL/6 mouse zygotes. Founder mice were identified through polymerase chain reaction (PCR) analysis of tail biopsy DNA using genotyping primers (mGpnmb-F and mGpnmb-R), which produced a 1,265-bp product for the knockout (KO) allele and a 6,871-bp product for the wild-type (WT) allele. Detailed sequences and positions are provided in the Supplementary Materials and Methods. The C57BL/6J WT strain was used as the control for animal experiments.

### CTX injections and cell isolation

A total of 40 μl of Naja pallida CTX (Merck KGaA), at a concentration of 10 μM in PBS, was injected into the tibialis anterior (TA) muscle of anesthetized mice Intramuscular injection (i.m.). Furthermore, to investigate the potential impact of rGPNMB on skeletal muscle regeneration, we simultaneously administered CTX at two concentrations (10 or 20 µg/kg) of rGPNMB. Injured TA muscles were collected at the indicated time points after injury. To examine the regenerating myofibers, 10-mm cross-sections were collected from the frozen TA muscles and stained with an embryonic myosin heavy chain (eMyHC, DSHB F1.652) antibody. The sections were imaged with a Nikon ECLIPSE Ti2 fluorescence microscope. Quantification of eMyHC staining was performed with ImageJ. Individual fibers were manually outlined to determine the cross-sectional area (CSA). At least 50 fibers per image and 3–5 images were analyzed at each indicated time point. We used a commercial murine skeletal muscle dissociation kit with a GentleMACS Octo Dissociator (Miltenyi Biotec). The TA muscles were excised and cut into small pieces following the manufacturer’s protocol. The digested product was filtered through a 70-µm cell strainer using a plunger to disrupt the undigested tissue and washed with RPMI-1640 containing 1% Penicillin/Streptomycin (P/S) and 20 mM HEPES and supplemented with 0.5% serum. After resuspension in 47% Percoll (Cytiva) and centrifugation at 1,500 rpm for 10 min, the cells were collected and washed for flow cytometric analysis or single-cell RNA sequencing (scRNA-seq).

### Magnetic separation of mononuclear cells and flow cytometric analysis

We used Magnetic-Activated Cell Sorting (MACS) separators to enrich the mononuclear cells obtained from the TA muscle to increase the number of macrophages. The mononuclear cells were isolated using anti-mouse CD90.2 and B220 MicroBeads (Miltenyi Biotec) through negative sorting per the manufacturer’s protocol to eliminate most T and B cells. Similarly, anti-mouse CD45 MicroBeads (Miltenyi Biotec) were used to sort the flowthrough. For flow cytometric analysis, we utilized TA muscle-derived mononuclear cells with a CytoFLEX flow cytometer (Beckman Coulter). In addition, fluorochrome-conjugated antibodies against CD45 (catalog 12-0454-82, eBioscience), CD11b (catalog 15-0112-82, eBioscience), CD68 (catalog 53-0681-82, eBioscience), Ly6C (catalog 17-5932-82, eBioscience) and GPNMB (catalog 50-5708-82, eBioscience) were used.

### Cell culture

Bone-marrow-derived cells (BMDCs) from mice were cultured according to a previous protocol^21^. In brief, BMDCs from the femur and tibia were flushed out using Dulbecco's Modified Eagle Medium (DMEM) and filtered through a 70-µm cell strainer. Red blood cells were removed using ACK lysis buffer (Thermo Fisher). BMDCs were cultured in DMEM supplemented with 10% fetal bovine serum, antibiotics (100 U/ml penicillin and 100 µg/ml streptomycin) and 20 ng/ml macrophage colony-stimulating factor (M-CSF; PeproTech) for 7 days to allow differentiation into BMDMs. The BMDMs were polarized into M1 or M2 macrophages by using Lipopolysaccharide (LPS) (100 ng/ml, Sigma Aldrich) plus IFN-γ (45 ng/ml, PeproTech) or IL-4 (10 ng/ml, PeproTech), respectively. C2C12 myoblasts were purchased from ATCC and cultured in DMEM supplemented with 10% FBS until confluency. After reaching confluency, the myoblasts were differentiated in DMEM supplemented with 1% horse serum for 72 h, as previously described^22^.

### Efferocytosis assay

An efferocytosis assay was conducted to evaluate the capacity of macrophages to phagocytose apoptotic cells in the context of GPNMB deficiency using an efferocytosis assay kit (Cayman). Mononuclear cells were isolated from the TA muscle on day 3 post-CTX-induced injury. C2C12 myoblasts were labeled with carboxyfluorescein succinimidyl ester (CFSE) to track their uptake by macrophages. Specifically, C2C12 cells were resuspended in assay buffer at 1 × 10^7^ cells/ml, then an equal volume of 2× CFSE Bait Cell Staining Solution was added for a final CFSE concentration of 5 µM. The cells were incubated at 37 °C for 30 min, washed three times with medium containing 10% FBS and induced to undergo apoptosis using staurosporine at a final concentration of 1 µM for 3 h. After inducing apoptosis, the CFSE-labeled apoptotic C2C12 cells were cocultured with BMDMs at a 1:1 ratio for 24 h to allow efferocytosis. After coculture, macrophages were stained with anti-F4/80 and anti-CD11b antibodies. Flow cytometry was then performed to quantify the percentage of macrophages phagocytosed apoptotic C2C12 cells, indicated by triple positivity for F4/80, CD11b and CFSE fluorescence. The rate of F4/80^+^CD11b^+^ macrophages containing CFSE^+^ material was compared between the GPNMB-KO and WT groups to assess the impact of GPNMB on macrophage efferocytosis.

### scRNA-seq (10x Genomics)

We isolated fresh cells and enriched them using MicroBeads to obtain high-quality scRNA-seq data, as previously described^23^. We then encapsulated these cells in droplet emulsions using a 10x Chromium Controller (10x Genomics) to achieve 10,000 cells per sample. The scRNA-seq libraries were prepared according to the manufacturer’s protocol using the GenCode Single-Cell 3′ Gel Bead and Library V3 kit. Subsequently, we pooled the libraries and sequenced them on a NovaSeq 6000 System (Illumina) following the manufacturer’s instructions.

### scRNA-seq data processing

We obtained 31,395 single cells with a median of 80,046 reads per cell. Paired-end scRNA-seq reads were demultiplexed, aligned to the mm10 reference genome and processed for single-cell gene counting using Cell Ranger Software from 10x Genomics (https://support.10xgenomics.com/single-cell-gene-expression/software). Downstream analysis of the combined sample gene counts was performed using Seurat, a scalable R-based package (version 4.3.0) designed for single-cell gene expression datasets. Gene counts were imported using the CreateSeuratObject function (min.cells = 25, min.features = 0), and low-quality cells were discarded using the following thresholds: a minimum of 500 and maximum of 5000 genes, a maximum of 10% of mitochondrial gene mapped reads, and a minimum of 1,000 and maximum of 40,000 unique molecular identifiers. The total number of cells that passed quality control according to the abovementioned thresholds was 21,642. SCTransform normalization, which uses regularized negative binomial regression for normalizing sparse single-cell data and variance stabilization, was performed for the filtered dataset using the SCTransform function in Seurat, regressing the percentage of mitochondrial genes per cell (vars.to.regress = “percent.mt”).

### Cell‒cell communication analysis

Cellular communication networks were quantitatively inferred and analyzed using scRNA-seq data. The open-source R package CellChat was used to visualize the interactions among different cell groups^24^. A total of 229 signaling pathway families were grouped as a library to analyze cell‒cell communication. Circle, hierarchy and river plots were generated according to the ligand‒receptor interaction network.

### Real-time qPCR

Total RNA was isolated from the TA muscle and C2C12 myoblasts with Direct-zol RNA Kits (Zymo Research), and mRNA levels for this study were determined by quantitative PCR on a CFX Opus 96 Real-Time PCR System (Bio-Rad). Primer 3 software was used across the intronic sequences to design all primers. The primers used for qPCR are listed in Supplementary Table 2.

### Statistical analysis

All the statistical analyses were performed with Student’s *t*-test or analysis of variance for comparisons of three or more groups using GraphPad Prism (GraphPad Software, V9.2.0). Differences were considered statistically significant when *P* < 0.05 (*P < 0.05, **P < 0.01, ***P < 0.001, ns: not significant).

## Results

### Dynamics of macrophage subset changes during skeletal muscle regeneration

**Fig. 1:**
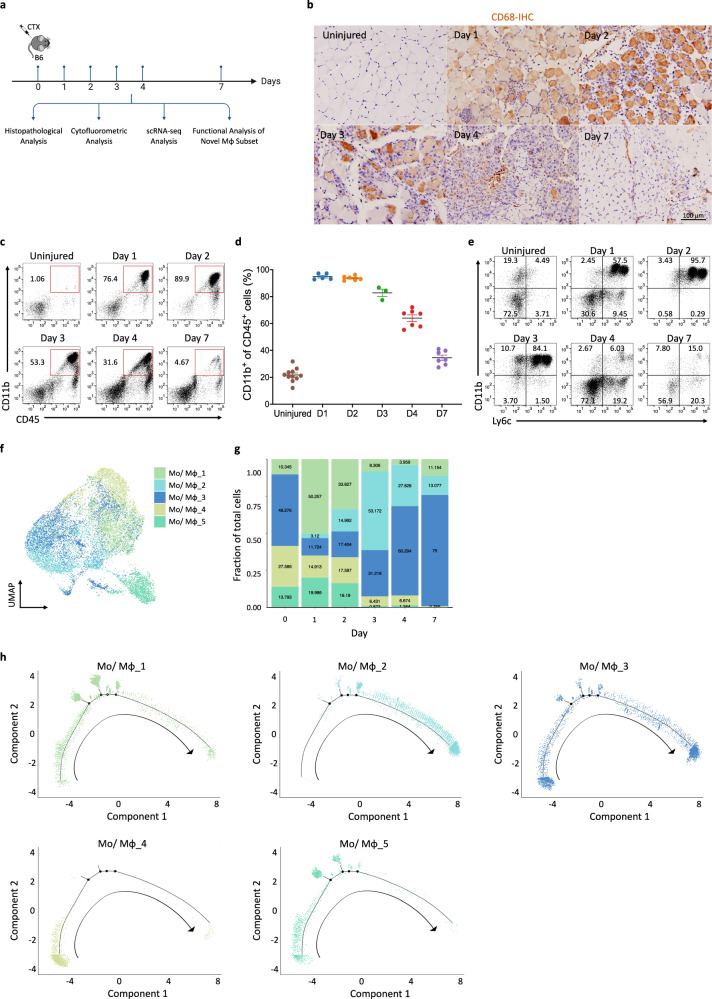
Identification of five distinct macrophage subsets during skeletal muscle regeneration *via* scRNA-seq. **a** A schematic showing the experimental timeline for CTX-induced skeletal muscle injury in mice, delineating the key analyses performed at various days after injury. **b** Immunohistochemical detection of Cd68^+^ macrophages within injured muscle at specified time intervals after injury. Scale bar, 100 µm. **c** Flow cytometric analysis showed a surge in CD11b^+^ cells immediately after injury, which diminishes starting from day 3. **d** A graphical representation of CD11b^+^ cell percentages, corresponding to flow cytometry results depicted in **c**. **e** The transition of macrophage phenotypes from proinflammatory Ly6c^hi^ early post-injury to anti-inflammatory Ly6c^lo^ by day 4, as assessed by flow cytometry. **f** A UMAP plot illustrating the distribution of five identified monocyte/macrophage subsets across the time series. **g** A bar graph showing the relative frequencies of each monocyte–macrophage subset at the designated time points after injury. **h** A pseudotime analysis for five monocyte–macrophage subsets, depicting the evolution of cellular states. The arrows indicate the proposed progression order through pseudotime, an analytical construct that aligns cells to represent their developmental continuum during muscle regeneration.

To reveal the changes in macrophage heterogeneity and identify the specific subsets influencing skeletal muscle regeneration following injury, we intramuscularly injected CTX into the TA of mice. The TA tissues were collected at the designated time points for multiple analyses (Fig. 1a). Consistent with previous reports^25^, these infiltrated immune cells were mainly CD68^+^ monocytes and macrophages (Fig. 1b). Cytofluorometric analysis confirmed the robust infiltration of circulating monocytes and the generation of macrophage subsets in the regenerating muscle. These cells were initially proinflammatory Ly6C^hi^ cells but transformed into anti-inflammatory Ly6C^lo^ cells by day 4 (Fig. 1c–e). For unbiased analysis that integrates temporal gene profiling of macrophages with analysis of their potentially varying roles in muscle regeneration, we enriched CD90.2^−^B220^−^CD45^+^ cell populations by using magnetic beads at the indicated time points during skeletal muscle regeneration. Subsequently, scRNA-seq analysis was performed using the 10x Genomics Chromium platform (Supplementary Fig. 1a). After standard quality control and the removal of doublets, high-quality transcriptomes from 21,642 cells were revealed. We performed graph-based Leiden clustering and utilized uniform manifold approximation and projection (UMAP) embeddings for visualization. All clusters were annotated by utilizing the scMCA_MNN-muscle dataset and differentially expressed genes (DEGs). Fifteen cell clusters were revealed, including five subsets of monocytes and macrophages (Mo/Mφ), five subsets of neutrophils and one subset each of dendritic cells, T cells, B cells, muscle progenitor cells and stromal cells (Supplementary Fig. 1b,c). As expected, macrophages and neutrophils were the most abundant cell populations after, with proportions of 67.9% and 15.3%, respectively (Supplementary Fig. 1c).

### Identification of five distinct macrophage subsets during skeletal muscle regeneration

We reclustered our scRNA-seq data of the macrophage subsets and identified five groups of macrophage subsets (Fig. 1f) whose distributions changed dynamically at different time points (Fig. 1g). Pseudotime analysis was performed on all five cell clusters along the injury-to-regeneration trajectory via Monocle (v3) to delineate the expression patterns of genes after muscle injury (Fig. 1h and Supplementary Fig. 2a). These dynamic changes suggest that the unique macrophage subset distributions may have critical biological functions at specific time points. The five distinct macrophage clusters (Mo/Mφ clusters: 1, 2, 3, 4 and 5) shared a common core of expressed macrophage markers, including *Adgre1*, *Cd68*, *Csf1r*, *Fcgr1*, *Lgals3* and *Lyz2* (Supplementary Fig. 1d). However, they exhibited distinct transcriptional profiles: cluster 1 exhibited increased expression of the M2 macrophage activation markers *Arg1* and *Mrc1*; cluster 2 exhibited increased expression of genes associated with tissue regeneration, such as *Igf1 and Gdf15*, which is in line with the recently described finding that GDF15 is a critical effector during skeletal regeneration^20^; and cluster 3 exhibited increased expression of genes associated with immune response activation and antigen presentation, including *H2-Aa*, *H2-Ab1* and *H2-Eb1*. Moreover, the proportion of cells in cluster 3 was notably greater than that of cells in other clusters in both the uninjured and regenerative stages; cluster 4 exhibited high expression of genes involved in proinflammatory responses, such as *Ifitm6*, *Gsr* and *Hp*; cluster 5 exhibited increased expression of *Acod1* (aconitate decarboxylase 1, also known as immunoresponsive gene 1), a key regulator of immunometabolism during infection and inflammation (Supplementary Fig. 1e). During the acute inflammatory stage (days 1 and 2), most cells were cells from clusters 1, 4 and 5, while there were only minor increases in the numbers of cells in clusters 3. With the transition from the acute inflammatory stage to the regenerative stage (day 3), the proportions of cells in clusters 1, 4 and 5 decreased, and there was a marked increase in the proportion of cells in cluster 2, which then declined by day 7. By contrast, the proportion of cells in cluster 3 exhibited a gradual and consistent increase over 7 days, peaking at the regenerative stage. Thus, our findings identified five macrophage subsets with dynamic changes throughout the regenerative process. Although the clusters 2 and 3 subsets were present in high proportions during the inflammatory-to-regenerative transition stage on day 3, cluster 2 cells expressed relatively higher levels of genes involved in macrophage-mediated tissue regeneration than did cluster 3 cells (Supplementary Fig. 1e). For example, IGF1 is one of the best-characterized growth factors and has been shown to regulate muscle regeneration^26^. IGF1 binds its receptor IGF1R to phosphorylate the intracellular adapter protein insulin receptor substrate-1 (IRS-1), which in turn activates the PI3K–AKT pathway to facilitate skeletal muscle regeneration. Moreover, ablation of triggering receptor expressed on myeloid cells-2 (TREM2), a major macrophage sensor known for supporting immune cell responses, has been noted to impede hepatic reparative processes in response to metabolic disruptions^27,28^. In view of these findings, it is conceivable that the macrophage cluster 2 we identified could play a role in facilitating muscle regeneration.

### A tissue-regenerative macrophage subset exhibiting a resident macrophage gene signature

Resident and recruited macrophages play distinct roles in immune defense, with resident macrophages providing a constant level of immune surveillance, while recruited macrophages respond to acute infections or injuries. To visualize differential gene expression patterns, we summarized the origin of each monocyte–macrophage subset and examined the known marker genes associated with resident and recruited macrophages^29^. Resident macrophage signature genes, such as *Axl*, *Cd74* and *Cxcl16*, were highly expressed in clusters 2 and 3, suggesting a resident-like gene profile. Clusters 4 and 5 were characterized by the expression of the recruited macrophage markers *Cxcr2*, *Ifitm1* and *Sell* (Supplementary Fig. 2). However, it is noteworthy that cluster 2 was conspicuously absent on day 0 (Fig. 1g), suggesting that cluster 2 cells may not be traditional resident macrophages. Our subsequent cell trajectory analysis suggests that clusters 4 and 5 may contribute to the formation of cluster 2 during the inflammatory-to-regenerative phase (Fig. 1h). These observations led us to hypothesize that, while clusters 2 and 3 display gene signatures typical of tissue-resident macrophages, they probably represent a transitional macrophage population that emerges during the inflammatory-to-regenerative phase of skeletal muscle healing. This hypothesis aligns with the notion that macrophage identity may be more fluid and context dependent than previously understood.

### Identification of GPNMB-expressing macrophages as critical effectors in skeletal muscle regeneration

**Fig. 2:**
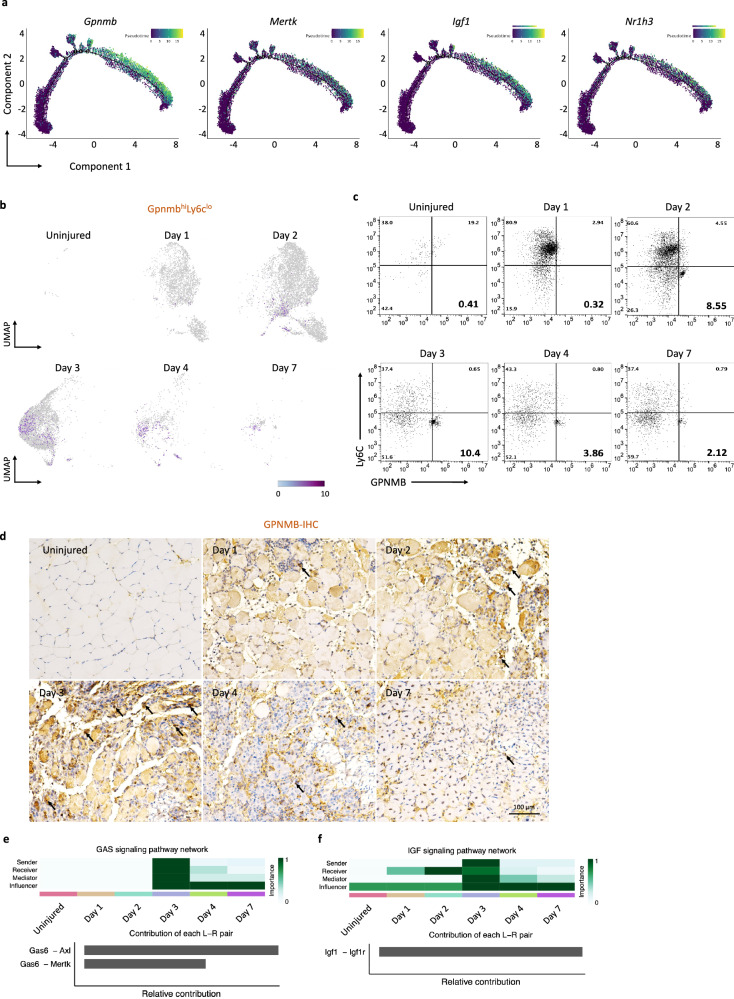
Temporal single-cell sequencing unveils GPNMB^+^ macrophage signaling as a dominant subset of tissue regeneration. **a** Trajectory plots tracing the gene expression profiles mirroring the *Gpnmb* pattern within the monocyte–macrophage subsets suggest synchronous gene expression events that may underpin shared functional pathways in regeneration. **b** UMAP series depicting the dynamic expression of the Gpnmb^hi^Ly6c^lo^ macrophage subset throughout the regenerative timeline. The UMAPs consecutively illustrate the prevalence and distribution of the Gpnmb^hi^Ly6c^lo^ population at each time point after skeletal muscle injury, highlighting the shifts in the macrophage landscape. **c** Flow cytometric quantification capturing the temporal prevalence of the Gpnmb^hi^Ly6c^lo^ population. The analysis delineates the frequency of this subset at progressive intervals post-injury, reflecting its involvement in the regenerative process. **d** Immunohistochemical staining of TA muscle sections across a temporal spectrum, identifying the presence of GPNMB-expressing cells. The arrows highlight GPNMB-positive macrophages in the injured regions, aiding in visualizing their cellular localization and temporal expression patterns during the regeneration phases. **e**, **f** Heatmap analysis summarizing the relative engagement of cell populations in GAS (**e**) and IGF (**f**) signaling pathways, offering insights into the predominant cellular functions at each time point (top) and detailing the contribution of specific ligand–receptor pairs to the composite signaling communication network (bottom).

A key analysis in the investigation of the molecular mechanisms underlying changes in the state of macrophages is the identification of DEGs along the pseudotime trajectory, that is, that determined by trajectory inference^30^ from scRNA-seq data. This inferred trajectory highlights the key effectors within macrophage subsets that govern the biological processes of regeneration. By integrating the relative trajectory positions of the macrophage clusters with the distribution density of each identified group (Fig. 1h), cluster 4 cells were identified as present mainly at the beginning of the trajectory; cluster 3 cells were predominant at both ends of the trajectory; cells in clusters 1 and 5 were identified at the early and middle positions; and cluster 2 cells were identified at the end of the pseudotime axis. Notably, the positions of cluster 3 cells at both ends of the pseudotime axis, together with their high proportions in uninjured muscle (Fig. 1g), suggest that cluster 3 may represent a subset with steady-state characteristics. To further investigate the biological and functional roles of cluster 2 macrophages during skeletal muscle regeneration, volcano plots were generated to visualize the DEGs in cluster 2 cells versus cells in clusters 1, 3, 4 and 5. The top 10 up- and downregulated DEGs are labeled in the plots; among these DEGs, the *Gpnmb* gene was significantly upregulated, specifically in cluster 2 (Supplementary Fig. 3). To further investigate the importance of macrophages with high GPNMB expression in tissue regeneration, we analyzed significantly activated macrophage marker genes^31^ using a pseudotime approach, focusing on critical factors involved in tissue regeneration and fibrosis. We found that *Mertk*, *Igf1* and *Nr1h3* exhibited the same expression pattern as *Gpnmb* (Fig. 2a); previous studies have highlighted the significance of these genes in tissue regeneration^26,32–34^.

In the advanced stages of tissue regeneration, macrophages adopt an anti-inflammatory phenotype that helps to suppress inflammatory responses and restore normal tissue structure and function. However, a dysregulated response can result in persistent inflammation and maladaptive regeneration, ultimately leading to tissue-destructive fibrosis. Previous studies have indicated that, in a chronic inflammatory environment, the GPNMB secreted by macrophages can stimulate excessive deposition of extracellular matrix, ultimately leading to pulmonary fibrosis^35^. After skeletal muscle injury, macrophages play a key role in clearing apoptotic cells and aiding tissue regeneration, a process that involves the conversion of infiltrating monocytes to macrophages with inflammatory and regenerative phenotypes. Our results revealed time-dependent gene expression changes in Gpnmb and Ly6c, revealing that the Gpnmb^hi^Ly6c^lo^ macrophage population was predominantly enriched in cluster 2 on day 3 (Fig. 2b). These findings highlight the importance of identifying specific GPNMB-expressing macrophage subsets during skeletal muscle regeneration. Based on the temporal dynamics of GPNMB expression and previous descriptions of tissue-resident macrophages^36,37^, we named this cell subset GPNMB^hi^Ly6C^lo^ ‘regenerative macrophages’. To validate the findings from scRNA-seq analysis, we used flow cytometry to assess the abundance of the GPNMB^hi^Ly6C^lo^ subset within the CD45^+^CD11b^+^ cell population in murine muscle after injury. We observed that this population peaked at 10.4% on day 3 before decreasing in abundance over the following days (Fig. 2c). Furthermore, our histological staining of tissue sections at various time points revealed prominent GPNMB-positive cells on day 3 (Fig. 2d). These findings suggest that macrophages with high GPNMB expression exhibit characteristics reminiscent of regenerative macrophages.

### CellChat identifies communication patterns and predicts the functions of macrophage subsets involved in skeletal muscle regeneration

As a direct comparison of DEGs might not comprehensively capture the intricate signaling network, we conducted a thorough investigation of macrophage cellular communication dynamics. We performed CellChat^24^ analysis at distinct time points and revealed regulatory cell‒cell interactions. On day 3, we observed increased levels of tissue regeneration-related signaling factors, including IGF^12,38^, GAS^39,40^, GDF^14^ and nicotinamide phosphoribosyltransferase (NAMPT)^7^. On day 3, macrophages tended to send signals of IGF and GDF and receive signals of IGF, GAS and GDF (Supplementary Fig. 4a). Inflammatory signals, such as IL-1, IL-2 and TNF in macrophages, were abundant on days 1 and 2 (Supplementary Fig. 4b). In addition to the signaling pathway network as a time-resolved signature, we also predicted the putative interactions among ligand and receptor pairs (Fig. 2e,f). These panels are based on the methodology described in Jin et al.^24^, where ‘sender’ refers to a cell secreting a signaling molecule, and ‘receiver’ refers to the cell activated by this molecule. ‘Relative contribution’ quantifies each component’s impact on the signaling network. On day 3, three critical skeletal muscle regeneration signaling pathways, GAS6–AXL, GAS6–MERTK and IGF1–IGF1R, were highly enriched. IGF1–IGF1R has been demonstrated to promote skeletal muscle regeneration^12,38^. The TYRO3, AXL and MERTK (TAM) receptor tyrosine kinases and their cognate glycoprotein ligands growth arrest-specific 6 (GAS6) and protein S (PROS1) are critical regulators of tissue homeostasis and inflammation^41^. Our results are consistent with the concept that TAM receptors are activated in macrophages in response to tissue injury^42^. The heightened activity of these three critical pathways confirms their roles in muscle regeneration and highlights their coordinated contribution to regenerative mechanisms.

### GPNMB promotes M2 macrophage polarization via the upregulation of specific transcription factors

**Fig. 3:**
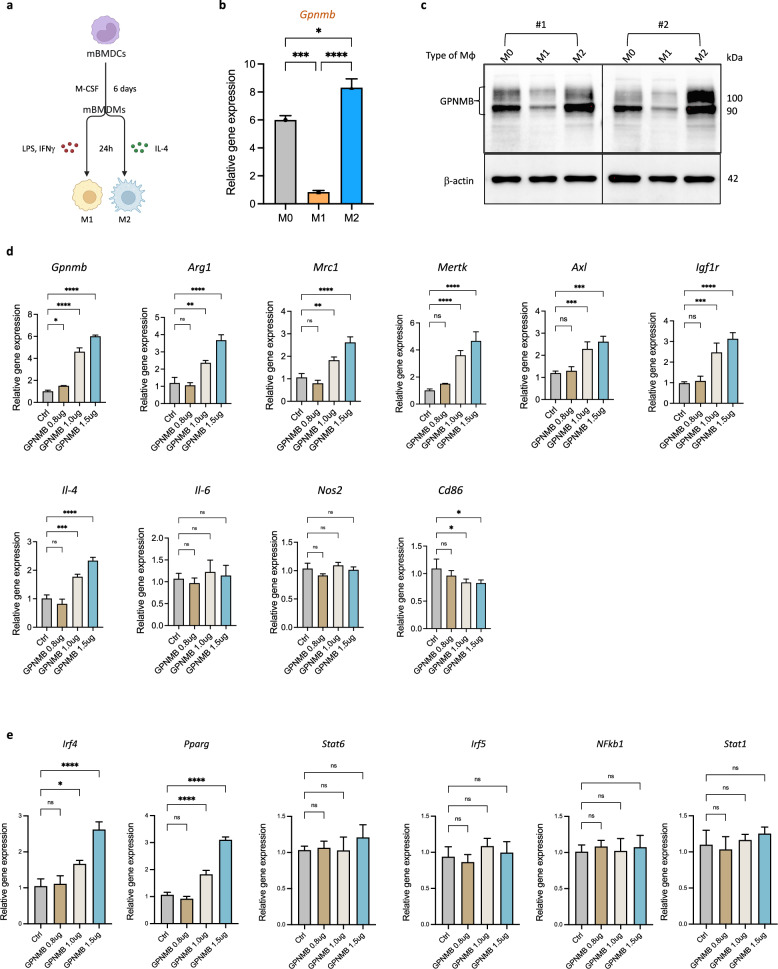
GPNMB overexpression enhances M2 macrophage polarization by modulating key regulatory genes. **a** The experimental scheme for differentiating murine BMDCs (mBMDCs) into M1 and M2 macrophages, followed by LPS, IFNγ and IL-4 treatment. **b** Quantitative PCR analysis shows significantly higher GPNMB expression in M2 polarized macrophages than in BMDM and M1. **c** Western blot analysis of GPNMB protein levels in BMDM, M1 and M2 macrophages from two WT mice (#1 and #2) confirmed the increased protein levels of GPNMB in M2 macrophages. **d** Overexpression of GPNMB in BMDMs leads to the upregulation of M2-associated genes *Arg1*, *Mrc1*, *IL-4*, *Mertk*, *Axl* and *Igf1r*, with no significant effect on the M1 markers *IL-6* and *Nos2*. **e** Overexpression of GPNMB results in heightened expression of M2-related transcription factors *Irf4* and *Pparg*, suggesting a potential pathway for GPNMB-mediated macrophage polarization.

To validate whether GPNMB can promote M2 macrophage polarization, we isolated murine BMDCs and induced their differentiation into M1 and M2 macrophages in vitro (Fig. 3a). mRNA and protein expression analyses revealed a significant increase in GPNMB expression in M2 macrophages compared with M1 macrophages (Fig. 3b, c). In addition, we investigated the effect of GPNMB overexpression on macrophage polarization. Our findings demonstrated that GPNMB overexpression significantly increased the expression of M2 macrophage-associated markers, including *Mrc1*, *Arg1*, *Mertk*, *Axl* and *Igf1r*, without affecting the expression of M1 macrophage-associated markers, such as *Il-6* and *Nos2* (Fig. 3d). These results indicate that GPNMB facilitates M2 macrophage polarization and serves as a marker of M2 macrophages involved in muscle tissue regeneration. (Fig. 3d). Specifically, overexpression of GPNMB significantly upregulated the expression of *Mertk*, *Axl* and *Igf1r* signaling pathway components, as shown in Fig. 3d. The overexpression of GPNMB was found to activate key transcription factors linked to M2 macrophage polarization, such as *Irf4* and *Pparg*. By contrast, the expression of the critical regulators *Stat6*, *Irf5*, *Nfkb1* and *Stat1* in BMDMs were unaffected by GPNMB overexpression (Fig. 3e), indicating that GPNMB may serve as a key regulator in the M2 gene expression program in macrophages.

### GPNMB KO impairs skeletal muscle regeneration

**Fig. 4:**
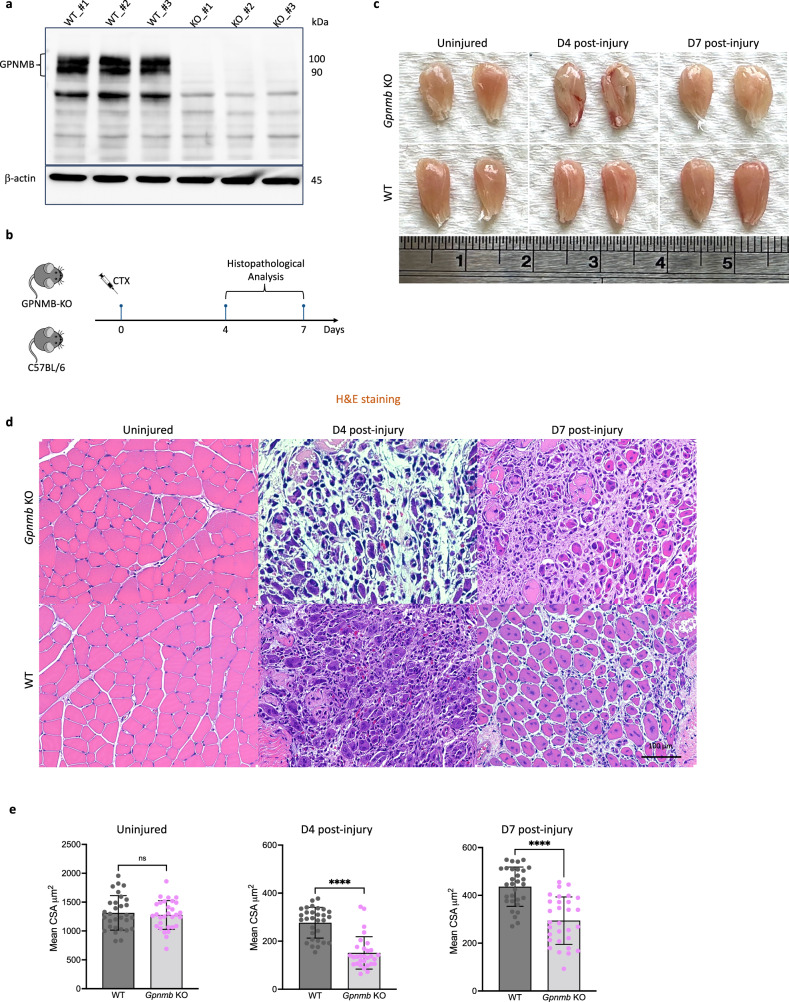
Impaired muscle regeneration in GPNMB-KO mice after CTX injury. **a** Western blot analysis confirmed the absence of GPNMB expression in GPNMB-KO mice compared with WT mice. **b** A schematic of the experimental design for assessing muscle regeneration in GPNMB-KO and C57BL/6 control mice after CTX injection. Mice were injected with CTX on day 0, and the TA muscles were collected for histopathological analysis on days 4 and 7 after injury. **c** Representative images of TA muscles from GPNMB-KO and WT mice that are uninjured and at days 4 and 7 after injury, demonstrating differences in muscle morphology. The GPNMB-KO mice exhibited impaired muscle regeneration with less striated muscle fibers than WT mice. **d** H&E staining of TA muscle sections revealed histological changes during regeneration, with GPNMB-KO mice showing reduced tissue repair compared with controls. **e** Quantitative analysis of CSA of muscle fibers in uninjured, day 4 post-injury and day 7 post-injury muscle sections confirmed statistically significant impairment of regeneration in GPNMB-KO mice.

To investigate the role of GPNMB in muscle regeneration, we generated GPNMB-KO mice and subjected them to CTX-induced muscle injury. Western blot analysis of muscle tissue extracts confirmed the absence of the GPNMB protein in the KO group, confirming that the observed phenotypic differences were attributable to GPNMB deficiency (Fig. 4a). The temporal evolution of muscle regeneration was monitored and compared with that of WT C57BL/6 controls (Fig. 4b). The gross morphological analysis of the TA muscles from the GPNMB-KO mice revealed marked impairment in muscle regeneration at days 4 and 7 after injury. The GPNMB-KO mice exhibited increased inflammatory cell infiltration in the injured muscle tissue, resulting in a visually apparent reddish discoloration and tissue edema compared to the WT mice (Fig. 4c). Histological evaluations conducted through hematoxylin and eosin (H&E) staining provided further insights into the compromised regenerative response in GPNMB-KO mice. Without GPNMB, the injured muscles exhibited exacerbated inflammatory responses and a notable impaired in myofiber regeneration (Fig. 4d). The injured sites in the GPNMB-KO mice were characterized by increased cellular infiltration and a lack of newly formed myofibers. This phenotype starkly contrasted with the organized regenerative patterns observed in WT controls. Quantitative analyses were performed to measure the CSA of muscle fibers, a key indicator of regenerative progress. On day 4 after injury, the mean CSA of regenerating myofibers in GPNMB-KO mice was significantly reduced, indicating a failure to initiate the regenerative process properly. This defect persisted through day 7, with KO mice displaying markedly smaller myofibers than WT mice (Fig. 4e). The statistical significance of these differences was confirmed, underscoring the necessity of GPNMB for efficient muscle regeneration. Our findings unveil a previously unappreciated role for GPNMB in facilitating muscle tissue repair, suggesting that GPNMB may act as a modulator of the cellular and molecular events that orchestrate the complex process of muscle regeneration after acute injury.

### Impaired macrophage efferocytosis and muscle regeneration after GPNMB KO and MERTK inhibition

**Fig. 5:**
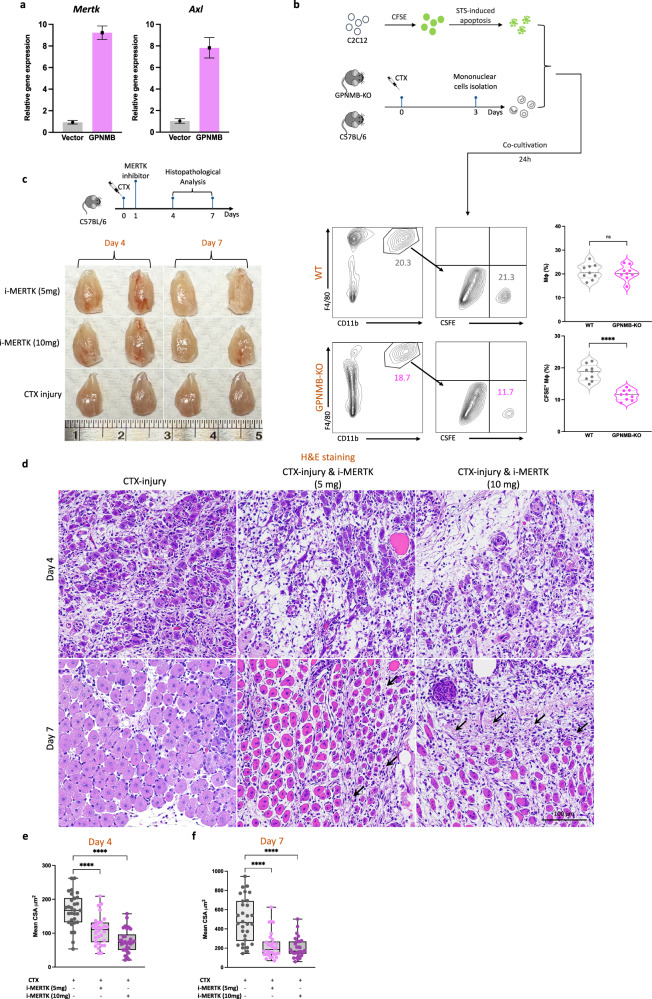
Diminished macrophage efferocytosis and impaired muscle regeneration after GPNMB KO and MERTK inhibition. **a** The experimental setup and subsequent flow cytometry analysis for evaluating macrophage efferocytosis. On day 3 after CTX-induced muscle injury, mononuclear cells were isolated from both WT and GPNMB-KO mice and cocultured with CFSE-labeled, staurosporine (STS)-induced apoptotic C2C12 myoblasts for 24 h. Flow cytometry was then used to assess the phagocytosis of apoptotic cells by CD11b^+^F4/80^+^ macrophages. The rightmost graph provides a quantitative comparison between the WT and GPNMB-KO groups, showing a significant decrease in efferocytosis efficiency in the GPNMB-KO macrophages, as reflected by their reduced uptake of CFSE-labeled apoptotic bodies. **b** Quantitative PCR analysis reveals that overexpression of GPNMB in primary macrophages significantly increases the mRNA levels of key efferocytosis genes, *Mertk* and *Axl*. **c** Left: a schematic of the experimental design depicting the treatment of C57BL/6 mice with a MERTK inhibitor after CTX injury to evaluate its effect on muscle regeneration. Right: the gross morphology of TA muscles from mice treated with MERTK inhibitor doses shows dose-dependent effects on muscle appearance. **d** Histological examination of muscle regeneration by H&E staining at days 4 and 7 after CTX injury with or without MERTK inhibitor treatment, highlighting the impact of MERTK signaling on tissue repair. **e**, **f** Statistical analysis of muscle fiber CSA and length at days 4 (**e**) and 7 (**f**) after injury, with MERTK inhibition leading to a marked reduction in both parameters, signifying compromised regenerative capacity.

GPNMB overexpression in primary murine macrophages led to the upregulation of the expression of efferocytosis-related genes, including *Mertk* and *Axl*, suggesting that GPNMB plays a regulatory role in the genetic orchestration of the efferocytosis process (Fig. 5a). We further investigated the functional role of GPNMB in macrophage-mediated efferocytosis. GPNMB-deficient macrophages exhibit a marked reduction in the phagocytosis of apoptotic cells. Fluorescence microscopy and flow cytometry analyses revealed that, compared with their WT counterparts, BMDMs from GPNMB-KO mice exhibited significantly decreased uptake of CFSE-labeled apoptotic C2C12 myoblasts, underscoring the importance of GPNMB in the clearance of apoptotic cells (Fig. 5b). To understand the broader implications of GPNMB deficiency for muscle regeneration, we further explored the impact of disruption of efferocytosis on muscle regeneration by using a pharmacological approach to inhibit MERTK. The administration of a MERTK inhibitor resulted in significant deficits in muscle tissue architecture and repair, as indicated by histopathological evaluations (Fig. 5c–f). Administering the inhibitor impaired muscle repair in a dose-dependent manner, as observed in histological analysis. (Fig. 5c). Histological analysis confirmed that higher doses of the MERTK inhibitor significantly compromised the muscle repair process on day 7 after injury. These morphological defects were confirmed by measuring muscle fiber CSA and length (Fig. 5d–f). These results underscore the importance of the GPNMB–MERTK axis in muscle regeneration and highlight a potential therapeutic target for promoting tissue repair after injury.

### GPNMB stimulation promotes muscle regeneration and the myogenic differentiation of murine myoblasts

**Fig. 6:**
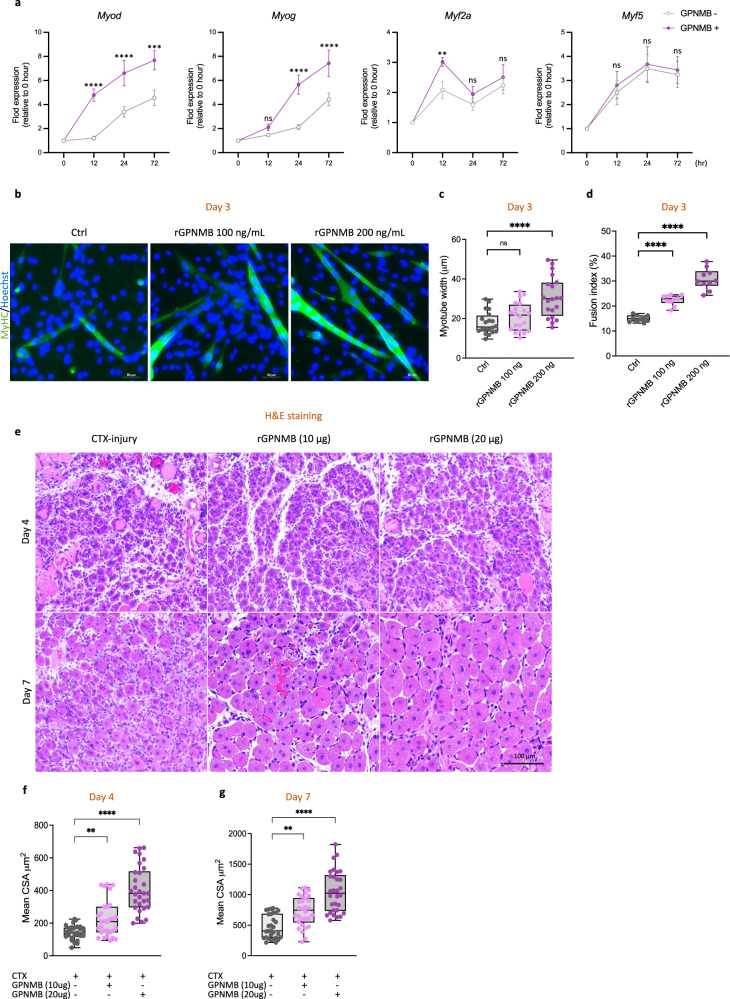
GPNMB stimulation promotes muscle regeneration and myogenic differentiation of murine myoblasts. **a** After GPNMB overexpression, quantitative real-time PCR was utilized to monitor the temporal expression of key myogenic regulatory factors in C2C12 cells, including *Myf5*, *Myf2a*, *Myod* and *Myog*. **b** Immunofluorescent staining for MyHC of C2C12 myoblast cultured in differentiation medium with (100 ng/ml or 200 ng/ml) or without rGPNMB. C2C12 myoblasts were induced to differentiate for 3 days. **c**, **d** Fusion indices were calculated by expressing the number of nuclei within MyHC-positive myotubes with ≥2 nuclei as a percentage of the total nuclei (**c**), and the myotube width was measured at 3 different points on the cell (**d**). The average width per myotube was calculated. Data are presented as mean ± s.d. of three independent experiments. **e** H&E staining of injured mouse TA muscle on days 4 and 7 with (10 µg/kg or 20 µg/kg) or without rGPNMB. Scale bar, 100 µm. **f**, **g** The CSA of myofibers from the injured-only group and the groups treated with rGPNMB (10 µg/kg and 20 µg/kg) on days 4 and 7. Data are presented as mean ± s.d. (*n* = 3) of each time point.

Previous research has indicated that the GPNMB expressed on macrophages undergoes enzymatic processing by disintegrin and metalloproteinase domain-containing protein 10 (ADAM10)^43^ to generate soluble GPNMB. Soluble GPNMB has critical functions; for example, it interacts with CD44 to promote cancer cell stemness and metastasis^44^. Via a similar pathway, soluble GPNMB promotes mesenchymal stromal cell survival, proliferation and migration^45^. Moreover, the binding of soluble GPNMB to syndecan-4 impedes the extravasation of activated T cells into inflamed skin^46^. To investigate the potential of GPNMB to promote myogenic differentiation of myoblasts, we performed myotube differentiation assays using C2C12 cells with or without the addition of rGPNMB. C2C12 cells treated with or without rGPNMB treatment were collected at several time points during the differentiation process, including 0 (before differentiation), 12, 24 and 72 h. The expression of *Myod* and *Myog* gradually increased during myoblast differentiation, and the addition of rGPNMB significantly increased their expression. However, rGPNMB only temporarily elevated the expression of the early differentiation marker *Myf2a* at 12 h and did not affect the expression of the myoblast proliferation marker *Myf5* at any time point (Fig. 6a). Immunofluorescence staining revealed that treatment with rGPNMB resulted in increased MyHC expression (Fig. 6b) and the formation of larger myotubes (Fig. 6c) containing a greater number of nuclei (Fig. 6d) in C2C12 cells than in the rGPNMB control group. Furthermore, our in vitro data indicate that rGPNMB facilitates myoblast differentiation. In vivo, we examined the role of exogenous GPNMB in facilitating skeletal muscle regeneration by administering 10 or 20 μg of rGPNMB to injured skeletal muscle. The results showed that the delivery of rGPNMB restored muscle architecture at the injury site (Fig. 6e). Specifically, on day 4, compared with injury alone, a single dose of rGPNMB led to a significant increase in the CSA of the TA muscle (225 ± 110.2 versus 128 ± 43.2 μm^2^, *P* = 0.019, in the 10 μg group; 408 ± 139.5 versus 128 ± 43.2 μm^2^, *P* < 0.0001, in the 20 μg group; Fig. 6f). Furthermore, on day 7, the effect of rGPNMB was sustained, as shown by the marked increase in the CSA of the TA muscle (728 ± 246.6 versus 471 ± 195.3 μm^2^, *P* = 0.0013 in the 10 μg group; 1051 ± 340.9 versus 471 ± 195.3 μm^2^, *P* < 0.0001 in the 20 μg group; Fig. 6g).

**Fig. 7:**
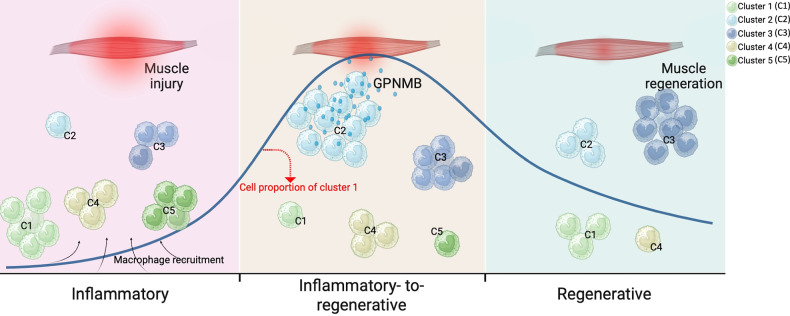
GPNMB-expressing macrophage contributes to skeletal muscle regeneration. The dynamic response of skeletal muscle to injury can be broadly categorized into three primary stages: the inflammatory, the inflammatory-to-regenerative and the regenerative stage. After muscle injury, muscle tissues recruit monocyte-derived macrophages, categorized into five distinct clusters (C1, C2, C3, C4 and C5) based on their inflammatory profiles. Our study identifies that macrophages with high GPNMB expression during the transitional phase from inflammation to regeneration are crucial in activating and expanding muscle progenitor cells. This process aids in the transition of macrophages toward an anti-inflammatory phenotype, which is critical for the resolution of inflammation and the subsequent muscle repair and regeneration. The dynamic shifts in macrophage subsets, highlighted by changes in the proportions of cluster 2, underscore the importance of GPNMB as an effector in muscle regeneration. Created with BioRender.com.

Our study delineates the crucial involvement of GPNMB-expressing macrophages in skeletal muscle regeneration. We identified five macrophage clusters recruited to the injury site, with cluster 2 macrophages notably expressing high levels of GPNMB during the inflammatory-to-regenerative transition. These macrophages play a pivotal role in activating and expanding muscle progenitor cells, facilitating the resolution of inflammation and promoting muscle repair. The dynamic shifts in macrophage subsets, particularly the increase in cluster 2, underscore the importance of GPNMB in the muscle regeneration process (Fig. 7).

## Discussion

Skeletal muscle regeneration is a complex process orchestrated by various types of cells, including MuSCs, FAPs and immune cells^1^. The contributions of macrophages to muscle regeneration promotion have been recognized for many years^9^. Recent studies have demonstrated that signals derived from macrophages, such as IGF1^12^, GDF15^14^ and NAMPT^7^, are effectors of skeletal muscle regeneration. In this study, we provide a temporal single-cell transcriptional characterization of macrophage subsets during skeletal muscle regeneration and highlight the heterogeneity of the macrophage population and its importance in the dynamics of muscle regeneration. We identified five macrophage clusters that differed in gene expression signatures and temporal dynamics during muscle regeneration. In particular, we identified a subset of macrophages characterized by elevated expression of GPNMB, IGF1, GDF15 and NAMPT. Furthermore, nutrient and oxygen availability, danger signals, antigens or instructional signals from other cells trigger changes in key metabolic regulatory events in immune cells. Previous studies have linked macrophage activation status to metabolic remodeling^47^, such as the enhancement of fatty acid oxidation and oxidative phosphorylation in M2 macrophages, which are crucial for M2 activation^48^. We found that cluster 2 macrophages exhibited markedly increased expression of M2 macrophage genes, including those involved in fatty acid oxidation, oxidative phosphorylation and the tricarboxylic acid cycle, such as PPAR-γ, PGC-1β and CPT2 (data not shown). These results suggest that high GPNMB expression in cluster 2 macrophages play a role in facilitating metabolic reprogramming to support M2 activation.

GPNMB has recently been reported to regulate macrophage inflammatory responses by inhibiting NF-κB signaling through its interaction with CD44^20^. However, the function of GPNMB varies among different tissue cells. For instance, liver-derived GPNMB binds to the CD44 receptor on white adipose tissue, leading to an increase in lipogenesis via the CD44–PI3K–mTORC pathway and resulting in obesity and insulin resistance^49^. Furthermore, the soluble form of GPNMB has been demonstrated to promote the recruitment of mesenchymal stromal cells, thereby promoting cutaneous wound healing^18,50^. In addition, growth factors such as IGF1 can promote skeletal muscle regeneration, and NAMPT can activate MuSCs through C–C motif chemokine receptor type 5. Here, we report a previously unrecognized subset of macrophages that contributes to muscle regeneration. Importantly, our CellChat results provided insights into the autocrine and paracrine mechanisms involving IGF1, GAS, GDF and NAMPT signaling interactions among macrophages at different stages of muscle regeneration.

Macrophages secrete factors that facilitate tissue regeneration by promoting the proliferation, differentiation and activation of various cell types, including stem and precursor cells. These cells adopt an anti-inflammatory phenotype during the later stages of tissue regeneration to suppress inflammatory responses and restore typical tissue structure. Dysregulation of this process can lead to persistent inflammation and maladaptive regeneration processes, ultimately resulting in tissue-destructive fibrosis^31^. Our RNA velocity analysis revealed the temporal dynamics of macrophages and identified *Gpnmb*, *Mertk*, *Igf1* and *Nr1h3* as pivotal indicators during skeletal muscle regeneration. These findings highlight the importance of GPNMB in this regenerative process. In response to efferocytosis, MERTK is activated by the intracellular modification of membrane cholesterol, yielding steroid metabolites that activate Nr1h3, which binds directly to the MERTK promoter to promote transcription^51^. Previous studies have shown that GAS6 can bind to phosphatidylserine, which is externalized on apoptotic cell membranes^52^, and activate MERTK on macrophages^6^; this provides positive feedback to further increase MERTK expression and ultimately shift macrophage polarization toward the M2 phenotype. Our results showed that the day 3 cell populations and cluster 1 subset have the characteristics of both receivers and senders of signals in the GAS6 signaling pathway. Interestingly, we observed that cluster 2, which infiltrates the wound site after day 1, partially exhibits the molecular signature of tissue-resident macrophages. This finding is important as it suggests that infiltrating macrophages can acquire characteristics of tissue-resident macrophages under specific conditions. Previous studies have shown that tissue-resident macrophages are crucial in maintaining homeostasis and promoting tissue repair. Our data indicate that cluster 2 macrophages may contribute to tissue regeneration by adopting a tissue-resident phenotype, potentially enhancing their regenerative capabilities. This dual role highlights the plasticity of macrophages and their ability to adapt to environmental cues, which is crucial for effective tissue repair. In vitro examination of the expression of GPNMB in murine M1 and M2 macrophages revealed that GPNMB was significantly upregulated in M2 macrophages compared with M1 macrophages, suggesting that GPNMB plays a role in M2 macrophage polarization. A gain-of-function assay was conducted to further validate that GPNMB overexpression promotes M2 polarization, indicating that GPNMB may serve as a macrophage M2 marker. In summary, our study revealed the importance of GPNMB-expressing macrophages, along with the genes *Mertk*, *Igf1* and *Nr1h3*, in tissue regeneration. Our study also revealed the significance of GPNMB-expressing macrophages and associated genes in tissue regeneration by revealing the gene expression timeline of regenerative macrophage subsets in injured muscle.

Muscle regeneration was indicated by significant increases in the CSA and multinuclear myofiber count in the TA muscle after the injection of rGPNMB. These results demonstrate the potential therapeutic role of GPNMB in promoting skeletal muscle regeneration. However, GPNMB may regulate multiple cell types involved in skeletal muscle regeneration in addition to infiltrating macrophages; these other cell types include FAPs and MuSCs. Further research is needed to elucidate the specific interactions and contributions of GPNMB^hi^Ly6C^lo^/regenerative macrophages and other cell types to the overall process of skeletal muscle regeneration. The findings presented in this single-cell study revealed the dynamics of macrophage subpopulation evolution during skeletal muscle regeneration and demonstrated the critical role of GPNMB^hi^Ly6C^lo^ regenerative macrophages in this process. Our analyses suggested that these macrophages secrete soluble GPNMB into the microenvironment to promote the proliferation, differentiation and maturation of MuSCs or myogenic progenitors. This research highlights the potential of GPNMB as a therapeutic target for promoting skeletal muscle regeneration and suggests a possible mechanism through which GPNMB exerts its effects. Given the limitations of our study, including the lack of lineage tracing, the assertion that clusters 2 and 3 originate directly from resident macrophages during muscle regeneration requires further experimental validation. This highlights a critical area for future research to delineate the origins and functional roles of these macrophage subsets in tissue repair and regeneration more definitively.

## Supplementary information

Supplementary Information
