# Supplementary material for: Temporal single-cell sequencing analysis reveals that GPNMB-expressing macrophages potentiate muscle regeneration
Source: Exp Mol Med. 2025 Jun 9;57(6):1232–45. doi: 10.1038/s12276-025-01467-4 (PMC12229484; doi:10.1038/s12276-025-01467-4)
Supplement: Supplementary file 1 — Supplementary Information [file 12276_2025_1467_MOESM1_ESM.pdf]

## Supplementary file list

1. Supplementary Materials and Methods
2. Supplementary Figures
3. Supplementary Tables

# Supplementary Materials and Methods

**Isolation and culturing of primary mouse macrophage**
Bone marrow-derived cells (BMDCs) were isolated from C57BL/6 mice. The collected cells were then incubated with ACK lysis buffer (Thermo Fisher Scientific) for 1 minute to lyse and remove red blood cells. The BMDCs were subsequently resuspended in DMEM growth media containing 2 mM l-glutamine, 100 units/ml penicillin, 0.1 mg/ml streptomycin, 10% FBS, and 20 ng/mL macrophage colony-stimulating factor (PeproTech). The cells were cultured for 6 days to become M0 macrophages. For macrophage polarization, M0 macrophages were either left unstimulated or stimulated overnight (16 hours) with LPS (100 ng/ml, Sigma Aldrich) and IFN-γ (10 ng/ml, PeproTech) to induce M1 macrophage differentiation, or IL-4 (10 ng/ml, PeproTech) to induce M2 macrophage differentiation. Macrophage polarization was further validated by assessing the expression of M1 markers TNF-alpha and IL-1beta and M2 markers CD206 and Arg1.

**GPNMB overexpression experiments**
To overexpress GPNMB in macrophages, the full-length GPNMB cDNA was cloned into the pCMV vector. BMDCs were cultured and differentiated into macrophages, as described above. On Day 7, the cells were transfected with the pCMV-GPNMB vector using Lipofectamine 3000 (Thermo Fisher Scientific) according to the manufacturer's protocol. The cells were then cultured for 24 hours to allow for GPNMB overexpression. Total RNA was extracted using the Direct-zol RNA Kits (Zymo Research) and reverse transcribed into cDNA using the iScript cDNA Synthesis Kit (Bio-Rad). Quantitative PCR (qPCR) was performed to assess the expression levels of GPNMB and other regulatory genes using SYBR Green Master Mix (Applied Biosystems) on a CFX Opus 96 Real-Time PCR System (Bio-Rad). The relative gene expression levels were normalized to Glyceraldehyde-3-phosphate dehydrogenase (GAPDH) as an internal control. The primers used for qPCR are listed in Supplementary Table 2.

**Western blot and immunohistochemistry staining**
Protein was extracted from cells and tissues using RIPA buffer (Thermo Fisher Scientific) supplemented with Mini Protease Inhibitor Cocktail (Merck) according to the manufacturer's protocol. Protein concentration was quantified using the Pierce BCA Protein Assay Kit. Proteins were separated on 10 or 12% SDS-PAGE gels (20 µg protein per lane) at 70 V and then transferred to polyvinylidene difluoride (PVDF) membranes (Millipore) at 300 mA. The PVDF membranes were blocked with Western Blot Buffers (Thermo Fisher Scientific) for 30 minutes and then incubated with a primary antibody solution at 4°C overnight. After washing the PVDF membranes with TBST three times, HRP-conjugated secondary antibodies were applied for a 1-hour incubation at room temperature. Chemiluminescent signals were visualized using the ECL Western Blot Detection Kit and Bio-RAD imaging system (Bio-Rad). Immunohistochemistry staining analysis was performed on mouse tibialis anterior (TA) muscle sections (10 µm) collected from frozen skeletal muscles using a cryostat. The sections were fixed in 4% paraformaldehyde (PFA) for 30 minutes, then washed in 1X phosphate buffer saline (PBS) and heated in citrate buffer (pH 6.0) for 45 minutes. The sections were maintained in a blocking buffer (10% goat serum diluted in 1X PBS) for 30 minutes. Slides were stained overnight at 4°C with primary antibodies diluted in staining solution (1% bovine serum albumin diluted in 1X PBS). The primary antibodies used for western blot and immunohistochemistry staining are listed in Supplementary Table 1.

**MERTK inhibition in murine muscle regeneration**
The MERTK inhibitor used in this study is UNC2025, a validated molecule known for its efficacy and specificity in inhibiting MERTK signaling[1]. C57BL/6 mice were administered the MERTK inhibitor for the experiments at concentrations of 5 mg/kg and 10 mg/kg. The inhibitor was delivered intraperitoneally starting one day post-CTX injury and continued for seven days to assess its impact on muscle regeneration. These concentrations and the method of administration were selected based on previous literature demonstrating the efficacy of UNC2025 in similar contexts. Histopathological analysis was performed at various time points to evaluate the effect of MERTK inhibition on muscle regeneration, with particular attention to inflammatory responses and myofiber regeneration.

**Generation of Gpnmb-KO mice using CRISPR-Cas9 technology**
To generate Gpnmb-KO mice, we employed the CRISPR-Cas9 genome editing system to delete exons 2 to 6 of the Gpnmb gene. The strategy involved designing two guide RNAs (gRNAs) targeting sequences flanking exons 2 to 6 of the Gpnmb gene. These gRNAs were combined with Cas9-RNPs and then microinjected into fertilized C57BL/6 mouse zygotes. The embryos were implanted into pseudopregnant female mice to generate founder mice. Genomic DNA was extracted from tail biopsies of the resulting pups, and PCR analysis was performed to screen for the desired deletion. The genotyping primers used were:
mGpnmb-F: 5'-CTGGGAGCATCCCCAAACTTTG-3'
mGpnmb-R: 5'-GGTTTAGAGTGAGATGAAGCTGTATGTCT-3'
The expected PCR product size for the wild-type allele was 6,871 bp, while the product size for the knockout allele was 1,265 bp, indicating successful deletion of exons 2 to 6. PCR products were then sequenced to confirm the precise deletion of the targeted region. Detailed sequences and positions are provided in Supplementary Materials and Methods Figure.

## Generation of Gpnmb-KO Mice Using CRISPR-Cas9 Technology

**a**

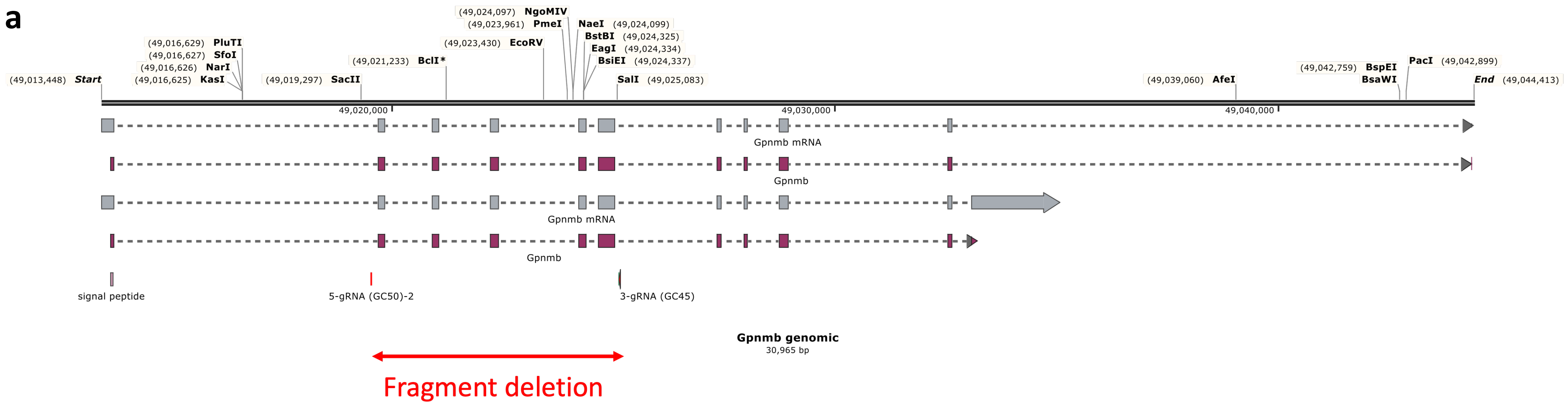


**b**

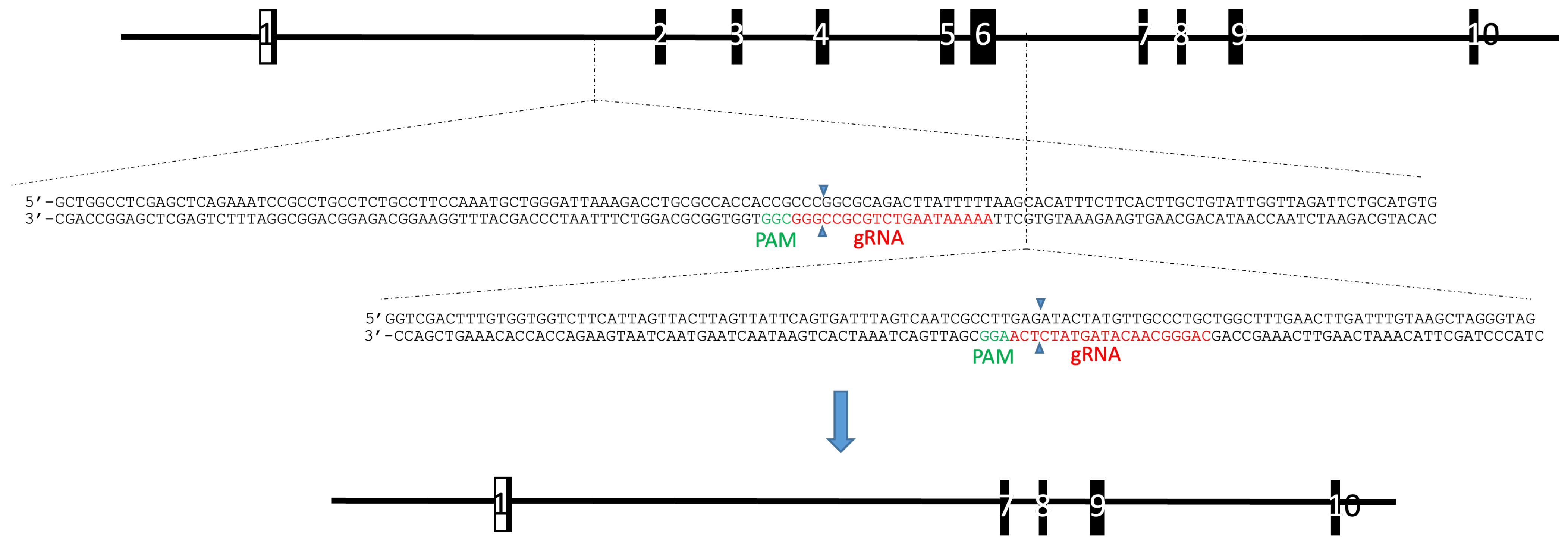


**c**

| | Genotyping primer |
|---|---|
| mGpnmb-F | CTGGGAGCATCCCCAAACTTTG |
| mGpnmb-R | GGTTTAGAGTGAGATGAAGCTGTATGTCT |

**Strategy for the generation of Gpnmb-KO mouse**

(a) Schematic representation of the Gpnmb gene targeting strategy using CRISPR-Cas9 technology. Exons 2 to 6 of the Gpnmb gene are targeted for deletion. The positions of the guide RNAs (gRNAs) are indicated. (b) A detailed sequence of the targeted region with the gRNAs and PAM sequences is highlighted. The fragment deletion between exons 2 and 6 is shown with the double-strand breaks indicated by red arrows. (c) Genotyping strategy using PCR. The sequences of the genotyping primers (mGpnmb-F and mGpnmb-R) are provided.

## Flow Cytometry Analysis

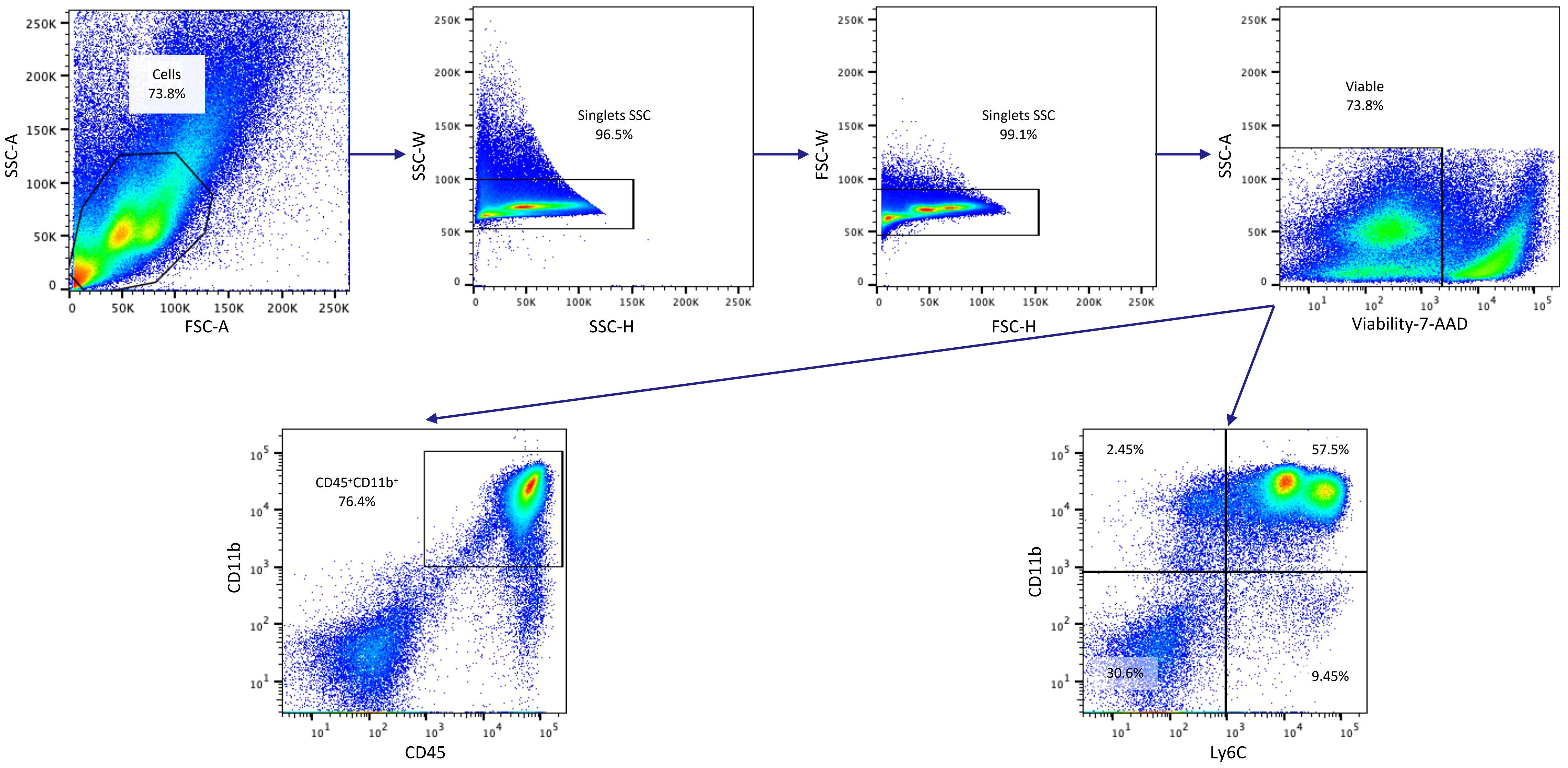


**Gating strategy for flow cytometry analysis**

Events corresponding to cells were gated on SSC-A vs FSC-A plots, then doublets were excluded. Events in singlets gate were further analyzed for the uptake of LiveDead Violet dye to exclude events corresponding to dead or damaged cells.

## REFERENCES:


1. Bae, SH., Kim, JH., Park, T.H. *et al.* BMS794833 inhibits macrophage efferocytosis by directly binding to MERTK and inhibiting its activity. *Exp Mol Med* 54, 1450–1460 (2022).

# Supplementary Figures

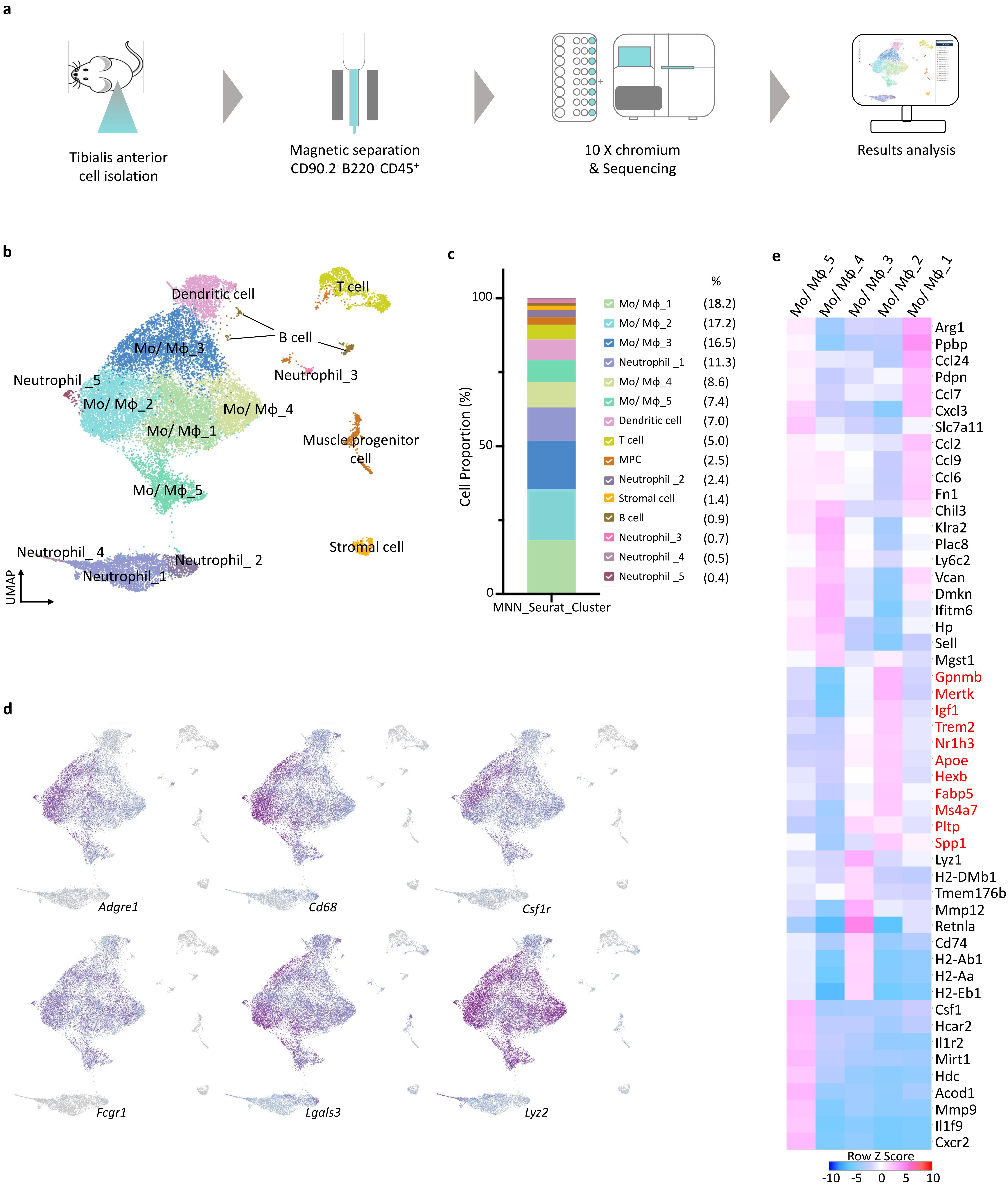


**Supplementary Fig. 1: Single-cell trajectory analysis elucidates monocyte/macrophage subset dynamics in muscle regeneration. (a)** Workflow illustration from the isolation of cells from the tibialis anterior muscle to single-cell RNA sequencing and data analysis. **(b)** UMAP plot of 21,642 single cells from the tibialis anterior (TA) muscle, post $CD90.2^{-}B220^{-}CD45^{+}$ selection, displayed post-unsupervised clustering into 15 distinct groups. Individual cells are represented as points and are color-coded by their cluster assignment. **(c)** Bar graph showing individual cell cluster proportion of total cells. **(d)** UMAP for the indicated monocyte/macrophage markers. **(e)** A heatmap details the differential gene expression patterns across the identified subsets. The red font annotations underscore genes significantly upregulated in Cluster 2. The color gradient represents the row Z-score, visually representing relative gene expression levels among the subsets.

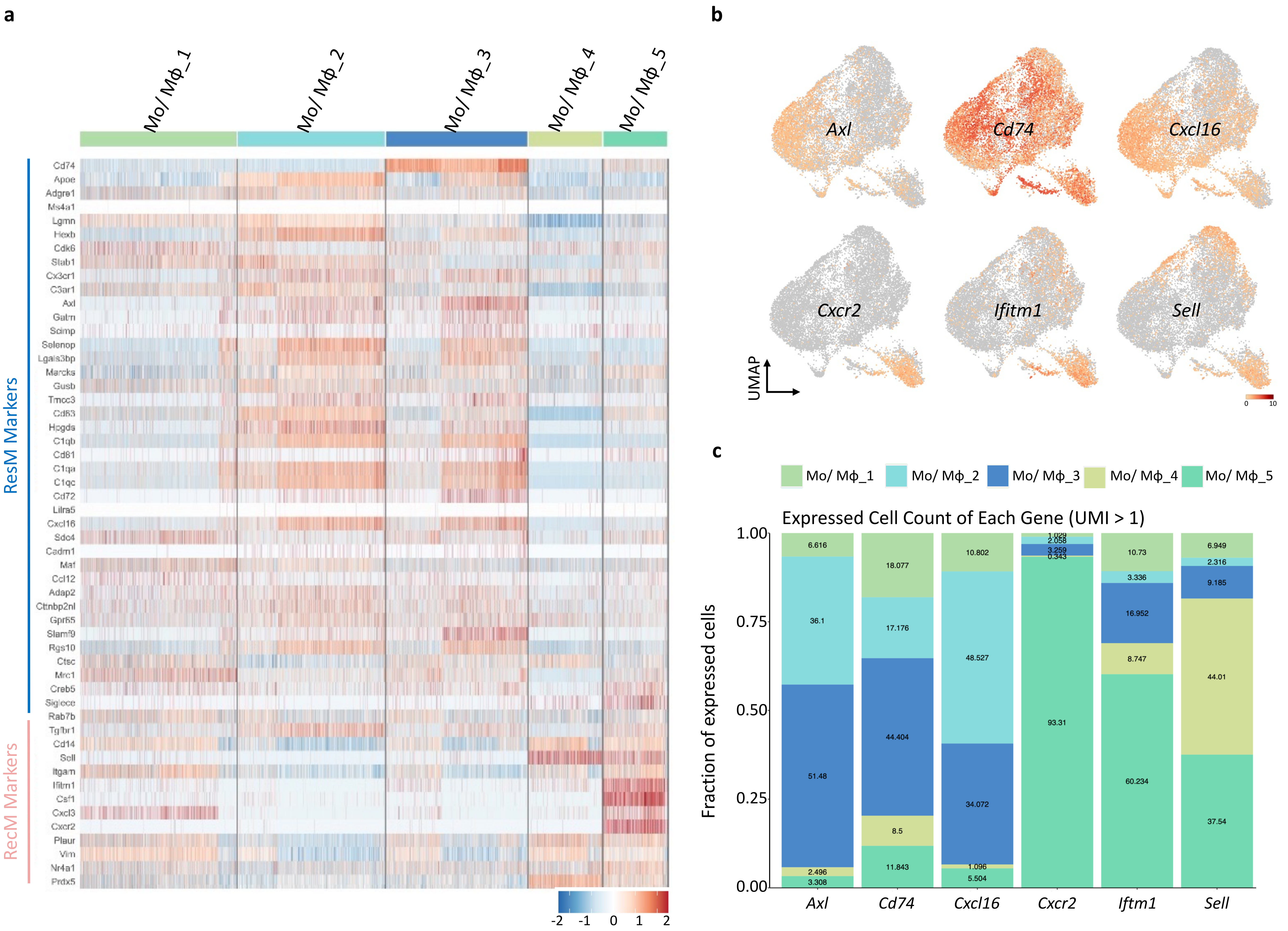


**Supplementary Fig. 2: Differential expression of resident and recruited macrophage markers across identified subsets. (a)** Heatmap representation of gene expression patterns reveals distinct profiles of resident and recruited macrophage markers within the five identified macrophage subsets, with Cluster 2 predominantly expressing resident macrophage signatures.

**(b), (c)** UMAP visualizations and quantitative expression data highlight the segregation of classical resident (*Axl, Cd74,* and *Cxcl16*) and recruited (*Cxcr2, Ifitm1,* and *Sell*) macrophage markers across the subsets.

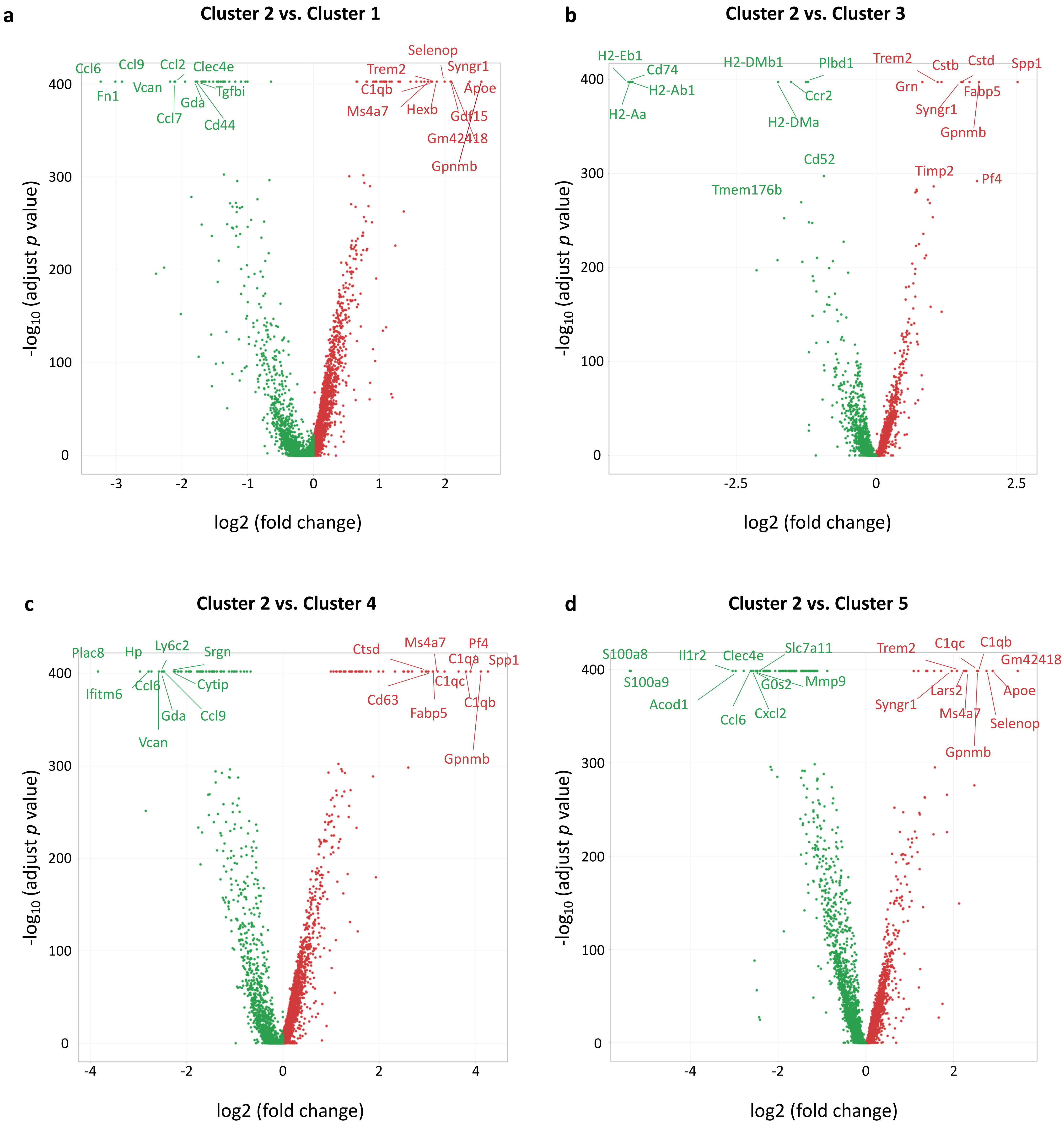


**Supplementary Fig. 3: Gene expression profiling highlights GPNMB as a distinguishing feature of monocyte/macrophage Cluster 2. (a)** Volcano plot comparing Cluster 2 with Cluster 1, showing upregulated genes (red) and downregulated genes (green) in Cluster 2. **(b)** Cluster 2 vs. Cluster 3. **(c)** Cluster 2 vs. Cluster 4. **(d)** Cluster 2 vs. Cluster 5. Each plot identifies the top 10 upregulated and downregulated genes in Cluster 2, emphasizing GPNMB as a key feature. Significant changes in gene expression are depicted by fold change (x-axis) and adjusted p-value (-log10, y-axis).

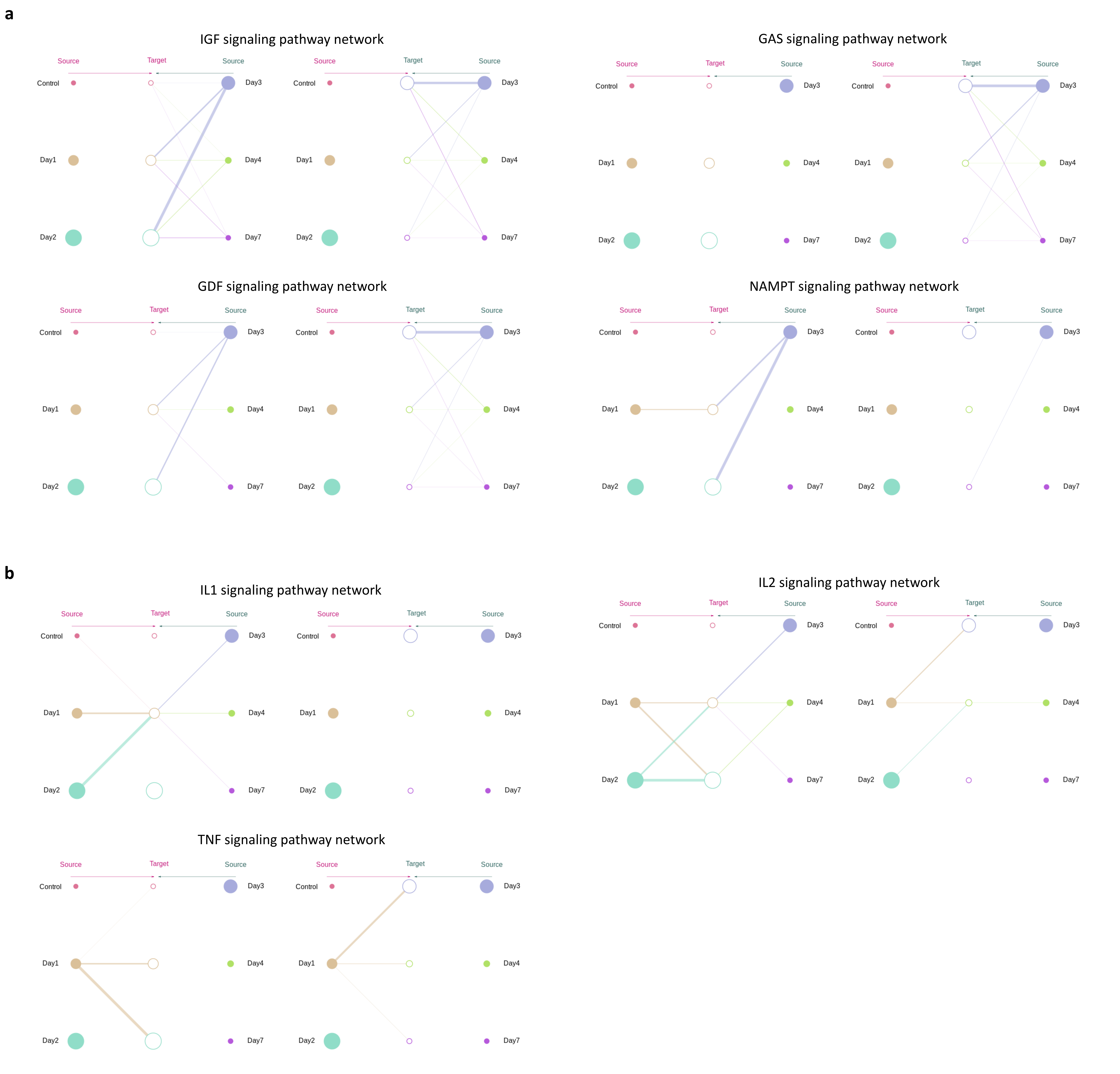


**Supplementary Fig. 4: Signaling network analysis of regenerative-associated macrophages. (a)** Hierarchical interaction plots demonstrate the dynamic signaling communication among macrophage populations over six-time points post-injury, mediated through IGF, GAS, GDF, and NAMPT pathways. The autocrine and paracrine signaling interactions are mapped to the corresponding time point trajectories, with circle sizes reflecting the number of cells in each group and line thickness indicating the probability of communication. **(b)** Early post-injury macrophages on day 1 and day 2 are characterized by their involvement in inflammatory signaling pathways, predominantly through IL-1, IL-2, and TNF. This network visualization underscores the shift in macrophage communication patterns from initial inflammatory responses to later regenerative signaling.

# Supplementary Tables

## Supplementary Table 1. Antibodies and microbeads

| Antibodies and reagents | Catalog no. | Regin | Species | purpose |
|---|---|---|---|---|
| Anti-CD11b antibody | 15-0112-82 | eBioscience | mouse | flow cytometry |
| Anti-CD45 antibody | 12-0454-82 | eBioscience | mouse | flow cytometry |
| Anti-F4/80 antibody | 48-4801-82 | eBioscience | mouse | flow cytometry |
| Anti-GPNMB antibody | 50-5708-82 | eBioscience | mouse | flow cytometry |
| Anti-Ly6C antibody | 17-5932-82 | eBioscience | mouse | flow cytometry |
| Anti-eMyHC | F1.652 | DSHB | human/mouse | IF |
| Anti-mouse IgG (H+L), F(ab')2 Fragment (Alexa Fluor® 488 Conjugate) | 4408 | Cell Signaling Technology | | IF |
| Hoechst | 4082 | Cell Signaling Technology | | IF |
| Anti-CD68 antibody | ab125212 | Abcam | mouse/rat | IHC |
| Anti-GPNMB antibody | 90205 | Cell Signaling Technology | mouse | IHC/WB |
| Anti-GAPDH antibody | 5174 | Cell Signaling Technology | human/mouse | WB |
| Anti-β-actin antibody | 4970 | Cell Signaling Technology | human/mouse | WB |
| Goat Anti-Rabbit IgG H&L (HRP) | ab6721 | Abcam | | WB |
| Goat Anti-Mouse IgG H&L (HRP) | ab6789 | Abcam | | WB |
| CD45R (B220) MicroBeads, mouse | 130-049-501 | Miltenyi | mouse | Cell separation |
| CD45 MicroBeads, mouse | 130-052-301 | Miltenyi | mouse | Cell separation |
| CD90.2 MicroBeads, mouse | 130-121-278 | Miltenyi | mouse | Cell separation |

## Supplementary Table 2. Primer sequences for real-time PCR

| Primer | (5' to 3') |
|---|---|
| mArg1-F | GAGGCCTATCTTACAGAGAAG |
| mArg1-R | GTGTTCACAGTACTCTTCACC |
| mAxl-F | GGTGTTTGAGCCAACCGTGGAA |
| mAxl-R | GCCACCTTATGCCGATCTACCA |
| mGpnmb-F | TGCTGTCTGTGAGAAGACCCTTC |
| mGpnmb-R | CAGGACACCATTCACTGCTCTCA |
| mIgf1r-F | CGGGATCTCATCAGCTTCACAG |
| mIgf1r-R | TCCTTGTTCGGAGGCAGGTCTA |
| mIl-4-F | CTCTAGTGTTCTCATGGAGCTG |
| mIl-4-R | CTTGGACTCATTCATGGTGCAG |
| mIl-6-F | GATGGATGCTACCAAACTGG |
| mIl-6-R | GGTAGCTATGGTACTCCAGA |
| mIrf4-F | AGACAGAGGAAGCTCATCACAG |
| mIrf4-R | GGTAACGTGTTCAGGTAACTCG |
| mIrf5-F | CAGTGTAAGGTGTTCTGGAGTG |
| mIrf5-R | CCTTCTGGAACAGGATGAGCT |
| mMef2a-F | TCTGTGACAACTCCGAGCTTG |
| mMef2a-R | CTTGTCCTAGATGGTGCTGCT |
| mMertk-F | GATTATTACCGCCAAGGCCGCATTGCC |
| mMertk-R | GTGGCCGTGGAGAAGGTAGTCGTACAT |
| mMrc1-F | CTTCTCTGGAATGCCTTCGAATG |
| mMrc1-R | GAGGTTCAACACGGTATGACAG |
| mMyf5-F | CAGCAGCTTTGACAGCATCTAC |
| mMyf5-R | GATGGCTCTGTAGACGTGATC |
| mMyod1-F | ACTACAGTGGCGACTCAGATG |
| mMyod1-R | TAGTAGGCGGTGTCGTAGCCATT |
| mMyog-F | CAATGCACTGGAGTTCGGTC |
| mMyog-R | CATATCCTCCACCGTGATGCT |
| mNf-κb-F | AACAATGCCTTCCGGCTGAGTC |
| mNf-κb-R | GATCACTTCAATGGCCTCTGTGTAG |
| mNos2-F | ATTGCTCCCTTCCGAAGTTTCTG |
| mNos2-R | CTTCCTGATAGAGGTGGTCCT |
| mPparγ-F | CATCCAAGACAACCTGCTGC |
| mPparγ-R | CATGTCTGTCTCTGTCTTCTTG |
| mStat1-F | AGAACCGATGGAGCTTGACGAC |
| mStat1-R | TCTGGAGACATGGGAAGCAGGTT |
| mStat6-F | GCTCAGATGTGACTATGGTAGAGG |
| mStat6-R | AGCTTGGTGAGGTCCTGTTCAGT |
